# Glycemic Responses of Milk and Plant-Based Drinks: Food Matrix Effects

**DOI:** 10.3390/foods12030453

**Published:** 2023-01-18

**Authors:** Blerina Shkembi, Thom Huppertz

**Affiliations:** 1 Food Quality & Design Group, Wageningen University & Research, 6708WG Wageningen, The Netherlands; 2 FrieslandCampina, 3800LE Amersfoort, The Netherlands

**Keywords:** dairy products, plant-based drinks, carbohydrates, metabolism, postprandial glycemic response, glycemic index, glycemic load, food matrix

## Abstract

The consumption of food items containing digestible carbohydrates in food products leads to postprandial increases in blood glucose levels and glycemic responses. The extent to which these occur depends on many factors, including concentration and type of carbohydrate, but also other physicochemical properties of the food matrix, which determine the rate of uptake of monosaccharides into the bloodstream, including product structure and factors affecting gastric emptying. For milk, control of postprandial glycemic responses appears to be multifaceted, including a controlled rate of gastric emptying, a rate of glucose and galactose uptake into the bloodstream controlled by enzymatic hydrolysis, as well as stimulated insulin secretion to enhance uptake of blood glucose from the bloodstream. Altogether, this allows milk to deliver comparatively high levels of carbohydrate with limited glycemic responses. For plant-based drinks positioned as milk alternatives, however, compositional differences (including carbohydrate type and concentration) as well as matrix factors limiting control over gastric emptying and insulin secretion can, in some cases, lead to much stronger glycemic responses, which are undesirable in relation to non-communicable diseases, such as type-2 diabetes. This review discusses glycemic responses to milk and plant-based drinks from this perspective, focusing on mechanistic insights and food matrix effects.

## 1. Introduction

Dairy products have traditionally made a strong contribution to the intake of essential nutrients in many countries. In the Netherlands, for instance, dairy products contribute almost 60% of total calcium intake, almost 40% of total intake of vitamin B2 and B12 and also more than 30% of the total intake of vitamin A and phosphorus and 20–30% of total intake of protein, zinc and iodide [1]. Dairy products also contribute to carbohydrate intake in the Netherlands, but only at ~10% of total dietary intake, which is below the contribution of dairy products to energy intake in the Netherlands (~15% of total energy intake) [1]. The carbohydrates from dairy are mainly in the form of lactose, a disaccharide consisting of galactose and glucose, linked by β-1→4 glycosidic bonds. Compared to other disaccharides, various benefits have been described for lactose, e.g., in relation to dental health, gut health and also glycemic index (GI) [2,3]. The lower GI of dairy products has been related to the fact that lactose is classified as a low-GI carbohydrate, but also due to the product matrix, particularly in relation to controlled gastric emptying [2,3].

In recent years, many plant-based food products have been introduced into the market and positioned as dairy alternatives, e.g., as an alternative to milk, yoghurt or cheese. These products may be prepared from legumes or nuts, from flours or may be composite products prepared from protein, fat and carbohydrate sources [4,5]. Products may be fortified with micronutrients, e.g., vitamins or minerals, and stabilizers may be added for mouthfeel and physical stability. While dairy products are regulated by nutrient standards in many jurisdictions, this is currently not the case for plant-based products. Recently, nutrient standards for plant-based drinks intended as milk alternatives have been proposed [6]. For proteins, the proposed standards included protein quantity but, for best-of-class products, also protein quality in required PDCAAS (protein digestibility-corrected amino acid score) values up to 0.9 and up to 0.8 for adults and children [6]. For sugars, however, only quantities were included in the proposed standards, with lower levels of sugars proposed for best-of-class products [6]. However, given the different glycemic responses from different carbohydrates, qualitative indicators may be required as well, particularly given that, for general products, proposed maximum carbohydrate levels [6] exceed those in milk, and carbohydrate type differs as well [7,8], entailing a risk of undesired glycemic responses.

This review focusses on the glycemic responses of dairy products and plant-based products positioned as dairy alternatives. For this, the background to postprandial glycemic responses will be described first, followed by the importance of the food matrix effect in determining glycemic responses. Subsequently, we will focus on the glycemic responses to consumption of dairy products and plant-based products. Differences observed will be discussed in the context of the food product matrix.

## 2. Postprandial Glycemic Responses

Glycemic response (GR) is the postprandial change in blood glucose concentration induced by the consumption of a carbohydrate-containing food or meal [9]. Postprandial glycaemia has been linked to the development of more diet-related diseases, such as obesity, type 2 diabetes mellitus and cardiovascular disease [10]. In healthy individuals, a normal fasting blood glucose concentration (no meals in the last 3–4 h) is generally between 80 and 90 mg/dL [11], whereas a level of 126 mg/dl or higher is associated with diabetes [12,13]. After a meal, blood glucose usually rises to 120–140 mg/dL and then gradually declines to the pre-meal values within 2 h [11]. Values higher than 140 mg/dL 2 h after food ingestion are defined as post-meal hyperglycemia [14].

**Figure 1:**
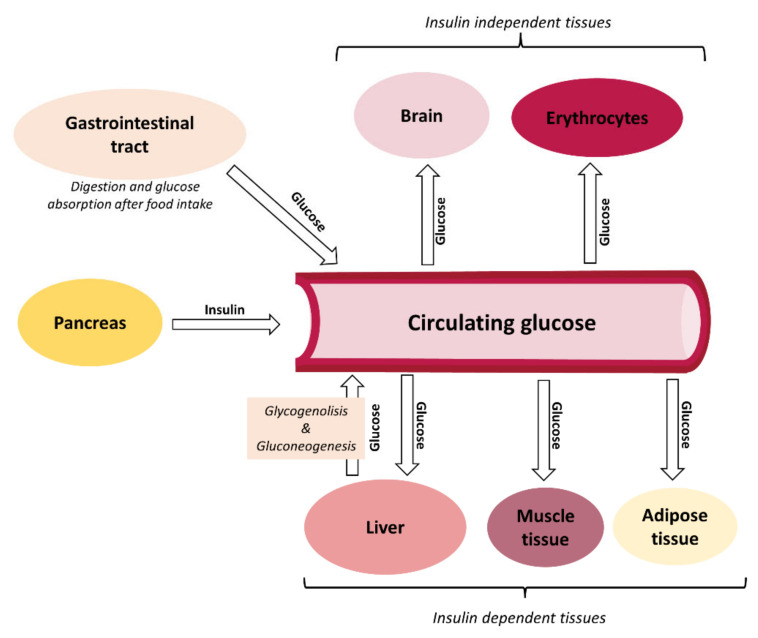
Schematic outline of postprandial glucose metabolism.

Blood glucose concentration is, as outlined in Figure 1, determined by the balance between the rate of glucose entering the circulation (glucose appearance) and the rate of glucose leaving the circulation (glucose disappearance) [15]. Circulating glucose comes from intestinal absorption during the fed state or, during fasting, from two metabolic pathways taking place in the liver: glycogenolysis (the breakdown of glycogen to glucose molecules) and gluconeogenesis (the generation of glucose from non-carbohydrate substrates) [15]. Glucose leaves the circulation via uptake by insulin-dependent (skeletal muscle, adipose tissue and liver) and insulin-independent (brain and erythrocytes) tissues and organs [16,17]. The major determinant of how rapidly glucose enters into the circulation during the feeding state is the rate of gastric emptying, the rate at which glucose passes from the stomach through the pyloric valve to the duodenum [15,18]. Rapid gastric emptying may lead to a considerable postprandial spike, while slow gastric emptying may reduce it [19].

Under physiological conditions, the blood glucose concentration is maintained within a specific range by the action of several hormones, including insulin, glucagon, amylin, glucagon-like peptide 1 (GLP-1), glucose-dependent insulinotropic peptide (GIP), epinephrine, cortisol and growth hormone (GH) [15,20]. Glucagon is a hyperglycemic hormone, secreted by the alpha cells in the pancreas in response to low blood glucose levels [15,20,21]. Glucagon plays an important role in the maintenance of blood glucose concentrations within a normal range during fasting, exercise and hypoglycemia by stimulating hepatic glucose production [15,22]. In the first 8–12 h of fasting, glucose is generated mainly through glycogenolysis and gluconeogenesis becomes the dominant mechanism of glucose production during longer periods of fasting [15,23]. Insulin is a hypoglycemic hormone produced by the beta cells in the pancreas as a direct consequence of high blood glucose levels [15,24], as well as in response to stimulation from GLP-1 and GIP (incretin hormones released from L and K cells in the intestine in response to nutrient/carbohydrate ingestion) [25,26,27]. In addition to stimulating insulin secretion from pancreatic beta cells, GLP-1 also inhibits glucagon secretion from pancreatic alpha cells but GIP does not inhibit it [15,25,26,27]. The main function of insulin is to maintain blood glucose concentrations within a normal range by increasing glucose uptake, utilization and storage in skeletal muscle and adipose tissue and decreasing glucose production from the liver (by decreasing gluconeogenesis and glycogenolysis) [15,24,28].

Postprandial glucose and insulin responses have been shown to be influenced by both the amount and source of carbohydrates consumed [29]. In 1981, Jenkins et al. [30] introduced the concept of the glycemic index (GI) defined as the incremental area under the curve (iAUC) for the blood glucose response over 120 min after the consumption of 50 g (or in some cases 25 g, if the portion is unreasonably large) of available carbohydrate portion of a test food expressed as a percentage of the corresponding area after the consumption of the same amount of available carbohydrates from a reference food (glucose or white bread) taken by the same subject on a separate occasion [30]. The GI is, hereby, set at 100 for the reference product, and the values of the test product are expressed relative to this. There can be differences in reported GI values based on the reference product used. GI values when using white bread as a reference are typically ~1.4-times higher than those obtained when using glucose as a reference [31,32]. Glucose would be more appropriate than the other reference foods (e.g., white bread), as the source of carbohydrates and product matrix is more standardized. In contrast, the composition and structure of white bread can vary from one experiment to another or from one location to another, making a comparison of the results from different studies more difficult [32]. Moreover, some studies have examined the impact of ethnicity on the glycemic response, reporting ethnic differences in postprandial blood glucose between Chinese people and Europeans [33,34], between Caucasians and non-Caucasians (for white bread) [35] and between Caucasians and Asians [36]. In contrast, some other studies demonstrated that the glycemic index is not influenced by ethnicity [37,38], but these conclusions are limited due to the low number of subjects involved in the studies.

Since the GI does not take into account the amount of carbohydrates consumed, the concept of glycemic load (GL) is also used [39]. The glycemic load (GL) is calculated based on the grams of available carbohydrates contained in a specified serving size and the glycemic index, using the following formula: GL = GI × available carbohydrates/100, where available carbohydrates (total carbohydrate—dietary fiber) can be expressed in different ways, such as gram (g) per serving, g per 100 g food, g per day’s intake and g per 1000 kJ or 1000 kcal, based on the context in which GL is used [9,40,41,42]. According to their glycemic load, foods are classified as follows: low GL (less than 10), intermediate GL (11–19) and high GL (up to 20) [41].

The GI of carbohydrates and carbohydrate-containing foods can be ranked based on their effect on postprandial glycemia [30]. High-GI products (GI ≥ 70) were classified as those that are digested and absorbed rapidly, causing an increase in the glycemic and insulin response after food ingestion [9,43,44]. Low-GI products (GI ≤ 55) are those digested and absorbed slowly, while medium-GI foods are those that have a GI between 56 and 69 [9,42,44,45]. It is generally considered that the more slowly the carbohydrate-containing food is digested and absorbed, the slower the blood glucose level rises after ingestion of food or meal [9].

Dietary carbohydrates are ingested in the form of long-chain polysaccharides (starch and fiber), disaccharides (e.g., lactose, sucrose, maltose and trehalose) and monosaccharides (e.g., glucose, galactose and fructose) but absorbed only in the form of monosaccharides [46]. Digestion of carbohydrates begins in the mouth through salivary α amylase, which breaks α→1,4 glycosidic bonds in starch and converts it into maltose (disaccharide) and other small polymers of glucose [47,48]. Salivary α amylase tends to be inactivated at the low pH (less than 3–3.5) in the acid gastric environment [49,50]. Carbohydrate digestion continues in the intestine, through the pancreatic α amylase, which digests around 60% of starch producing maltose, maltotriose and α-limit dextrins [48,49,51]. The digestion process is completed at the level of the cell membranes of the intestinal mucosa by other enzymes, such as lactase (which breaks lactose into glucose and galactose), sucrase (which breaks sucrose into glucose and fructose) and maltase (which breaks maltose into two glucose molecules) [48,49,52]. After the carbohydrates are broken down into monosaccharides (glucose, galactose and fructose), they are transported inside the intestinal cells through specific protein transporters present in the apical and basolateral membrane of the cell [49,50]. The most abundant dietary monosaccharide absorbed is glucose, followed by galactose and fructose [47]. From the intestinal cells, glucose, galactose and fructose are transferred into the circulation and then transported through the portal vein to the liver [49,50,53]. The liver converts most of the fructose and almost all of the galactose into glucose, which can be released through the liver cell membrane back into the circulation [48,54]. In addition to carbohydrates, other food factors that affect the glycemic index include the particle size, preparation, cooking and food processing, the physical form of the food and also the presence of other macronutrients [55,56]. High amounts of protein or fat present in a carbohydrate meal have been shown to reduce the glycemic response (through similar mechanisms) by slowing gastric emptying and increasing insulin secretion [57,58,59]. From such studies, it is clear that viewing glycemic responses goes beyond just carbohydrate concentration and type and needs to consider complete product composition. In fact, it should go even further and consider specific food matrices, as is discussed in Section 3.

## 3. Postprandial Glycemic Responses: Food Matrix Effects

As outlined in Section 2, the rate at which glucose and other monosaccharides enter the bloodstream is a key determinant of blood glucose levels and the manner in which the body responds to these. In turn, the rate of gastric emptying of the carbohydrates is a key factor affecting this. When pure carbohydrate solutions are consumed, there is no real inhibitors of gastric emptying except for a caloric and volume-flow restriction [60]. However, when looking at foods, rather than isolated components, all other constituents of the food, as well as their interactions, which can result in particular physical structures, need to be considered. Such holistic approaches, wherein the whole food is considered, rather than reductionistic single-component approaches, have gained a lot of traction in food and nutrition research and effects found as a result thereof are typically denoted as food matrix effects [61,62,63]. Food matrix effects have been described for postprandial lipidemia [64,65], aminoacidemia [66,67] and for bioavailability of micronutrients, e.g., calcium [68] and zinc [69], but also for postprandial glycemic responses.

One of the earliest studies clearly demonstrating a food matrix effect was on postprandial glycemic responses of apple products by Haber et al. [70] who showed that plasma glucose and serum insulin levels after consumption of apples differed markedly based on whether the apples were consumed as whole apples, apple puree or apple juice. Such effects can be related to the fact that, in apples, the carbohydrate is entrapped in cell structures, which need to be broken down prior to release from the stomach, as well as the presence of fiber, which slows down carbohydrate digestion. A similar importance of food matrix for fruits was also reported by Bolton et al. [71], who found that orange consumption leads to a lower insulin response to the whole fruit than orange juice and to a lower post-absorption plasma glucose drop, suggesting that the glycemic and insulin responses of the whole fruit depend on both the glucose and the fibers contained in the fruit [71]. Tey et al. [72], comparing different forms (bite size, puree) of two fruit types (guava, papaya) in 19 healthy elderly and young adults, found that the shape of the fruit influences the glycemic response in both elderly and young adults. Although all types of fruit had a lower glycemic index, guava puree and papaya puree gave significantly higher glycemic indices than guava bites and papaya bites [72].

These results are in line with the aforementioned studies where a lower plasma glucose value was found after the consumption of whole fruit with a larger particle size than that of fruit juice.

In addition to fruits, effects of macroscopic product structure on glycemic responses have also been tested for other food types, e.g., for lentils in various forms [73]. Further, in this case, more intensive processing of the lentils prior to consumption resulted in stronger increases in postprandial blood glucose levels [73]. Reynolds et al. [74] demonstrated the importance of food structure on glycemic control in adults with type 2 diabetes comparing four types of wholegrain bread with different particle sizes. As a result, the consumption of wholegrain bread made with more intact, coarsely ground whole grains lowered postprandial blood sugar in adults with type 2 diabetes compared to wholegrain bread made with finely ground whole grains. These findings, that glycemic responses to bread intake are governed by matrix effects, are in line with previous findings by Jenkins et al. [75] that blood glucose levels rose considerably more extensively after consumption of white bread from gluten-free flour than from regular flour. From these studies, and many more in the field, it is clear that glycemic responses are dependent not only on carbohydrate type, but also very much on the food matrix. This is also the case for dairy products and plant-based products positioned as dairy alternatives, as will be discussed in detail in subsequent sections.

## 4. Glycemic Responses of Dairy Products

When considering dairy products in relation to glycemic responses, some interesting effects are observed, which can be related to food matrix effects on various levels. The main carbohydrate in dairy products is the fermentable carbohydrate lactose, a disaccharide composed of galactose and glucose coupled by a β-1→4 glycosidic bond [2]. The β-1→4 glycosidic bond is rather unique among dietary carbohydrates, which typically contain and α-1→4 glycosidic bond [76]. The latter is susceptible to hydrolysis by glycosidic enzymes from a large number of micro-organisms, including, e.g., those in the dental microflora [77,78]. The resultant monosaccharides can be fermented by the dental microflora, leading to acid production and concomitant dental demineralization [77,78]. Because of the β-1→4 glycosidic bond, lactose is not readily hydrolyzed by the glycosidic enzymes from the dental microflora, making it the least cariogenic fermentable dietary carbohydrate [79,80,81]. The β-1→4 glycosidic bond of lactose is susceptible to hydrolysis by galactosidases, such as those in the small intestine, but also those of the lactic acid bacteria typically used in the production of fermented products, such as yoghurt, cheese and fermented milk. In these products, part or all of the lactose is converted into glucose and galactose, which can subsequently be fermented to lactic acid. Milk typically contains 4–5% lactose, whereas in yoghurt, 20–30% of lactose is fermented. In most cheese varieties, such as Cheddar and Gouda, little or no residual lactose is found [82].

The glycemic index of lactose has been determined and is reported as 46, which classifies it as a low-GI carbohydrate. Interestingly, the monosaccharides of lactose, galactose and glucose have reported GI values of 23 and 100, respectively [3], so the value of lactose is lower than what would be expected based on its constituent monosaccharides. In contrast, for sucrose, the reported GI value of 65 is very much in line with expectation based on the GI values of glucose (GI = 100) and fructose (GI = 23). The lower GI value of lactose compared to what would be expected based on its monosaccharides is related to the slower hydrolysis and absorption of lactose compared to sucrose, thereby modulating the rate of uptake and enhancing the proportion of glucose extracted from the blood after absorption [83,84]. In this way, enzymatic activities in the body control the glycemic response of lactose. Such control systems do not appear to be present for sucrose or other saccharides, whose hydrolysis is notably quicker [83,84].

While lactose alone is, thus, already classified as a low-GI carbohydrate, GI values of dairy products are even lower than what would be expected purely based on lactose content. Glycemic index, insulinemic index and glycemic load values of dairy products obtained from various in vivo studies are shown in Table 1. For example, Atkinson et al. [40] reported the average glycemic index of 62 common foods (obtained from multiple studies of various laboratories), including dairy products. A low GI was observed in all dairy products ranging from 37 to 51. Furthermore, Henry et al. [85] determined not only the glycemic index but also glycemic load of a variety of foods commercially available in the UK and found a low GI and GL values for all milks (GI ranged between 25 and 48; GL ranged between 3 and 6) and also for natural low-fat yoghurt (GI= 35; GL = 4). Hoyt et al. [86] reported a low GI value and a high insulinemic index (II) in both skimmed and whole milk, indicating that some components present in milk are able to stimulate insulin secretion [87]. Furthermore, Jenkins et al. [30] found a low glycemic index not only in skimmed, whole milk and yoghurt but also in ice cream (32, 34, 36 and 36, respectively). Östman et al. [87] analyzed the glycemic and insulinemic responses in ten healthy subjects after intake of regular milk and fermented milk products (Filmjölk and Ropy milk). Furthermore, a lactose solution (prepared with pure lactose and water) was included before each test meal. A low glycemic index (15–30) and a high insulinemic index (90–98) were found in all milk products, whereas for the lactose solution, a higher glycemic index and lower insulinemic index were found compared to milk products.

The insulinotropic effect of milk products can probably be attributed to amino acids and lipids, known to increase insulin secretion or the insulin requirement of a meal [87]. Anafy et al. [88] analyzed, in twenty healthy adult volunteers, the glycemic responses after intake of bovine milk-protein-based and lactose-free commercial infant formulas, containing lactose or glucose syrup solids instead of lactose but with the same protein composition (whey protein 60% and casein 40%). A low and similar glycemic index value was observed in both formulas (GI = 21.5), and the postprandial insulinemic response, during the two-hour period, was similar. Various studies suggest that dairy consumption in particular yoghurt consumption is associated with a reduced risk of type 2 diabetes, given the low glycemic index, largely attributable to dairy nutrients, as explained above [42,89,90,91,92]. In a study by Wolever [42], the glycemic index value of 43 plain or artificially sweetened yoghurts was compared with that of 50 sweetened yoghurts (the GI values were taken from the GI database of the University of Sydney), and it was found that all products had a low GI but the GIs of plain or artificially sweetened yoghurts were lower than those of sweetened yoghurt (with an average of 26.6 vs. 40.5). This difference is probably due not only to the level of sugars, but also to the higher protein to carbohydrate ratio in plain yoghurt. Furthermore, it is interesting to note from this study that even sweetened yoghurt could be classified as low GI.

The lower GI value in milk compared to pure lactose (Table 1) may be related to the fact that gastric emptying for milk is notably slower. This can be related to a number of reasons. First, milk contains other macronutrients than lactose and, with gastric emptying to a notable extent determined by caloric restrictions, the amount of lactose emptied from the stomach to the intestines per time-unit will be smaller [60]. Particularly undenatured whey proteins, which are present in milk at levels of approx. 0.7%, will also empty rapidly from the stomach [93,94]. On the other hand, caseins and fat are known to empty notably slower due to gastric coagulation. In addition to providing additional caloric value to the gastric digesta being emptied, whey proteins also play another important role. Whey proteins have been shown to reduce gastric pH levels through increased concentrations of GIP and GLP-1. Furthermore, whey proteins are also known to stimulate secretion of incretin hormones, which further exerts glycemic control [95,96,97]. A further aspect to consider is the buffering capacity of milk. When an adult consumes a serving of milk, gastric pH increases to up to 6 [98] and substantial amounts of gastric fluid are required to bring back pH to the initial value of pH ~2. It has been estimated that this requires approximately an equal volume of gastric fluid, relative to the volume of milk consumed [68]. This dilution reduces lactose content in the gastric content and can further decrease emptying of lactose and subsequent hydrolysis and uptake of glucose and galactose. From the above, it is clear that synergy between the chemistry and physicochemical properties of milk and the human body leads to an excellent control of glycemic response, allowing milk to deliver a sizeable amount of energy in the form of lactose without resulting in excessive rises in blood glucose level. Such control mechanisms are perhaps not surprising, considering the role of milk as the sole source of nutrition for neonates.

What is also interesting to consider, in this respect, is that milk is the only liquid food product that can be consumed in the form of the whole food produced as a primary commodity. All other liquids consumed in the human diet (with the exception of water) require one or more prior conversion steps from semisolid products to liquid products, e.g., the juicing of fruits or vegetables or the extraction of carbohydrates from crops and their subsequent use in a variety of beverages. In all these cases, physical control mechanisms enabling controlled-release carbohydrates from these products are diminished, as is apparent from examples on producing juice from apples [70] and oranges [71], highlighted in Section 3.

Milk is also converted into a number of other products, most notably cheese, yoghurt and fermented milk. In cheese, (virtually) all lactose is removed or fermented during production and ripening, but in yoghurt, substantial amounts of carbohydrate remain, as only 20–30% of lactose is fermented in the manufacture of yoghurt [82,99]. Glycemic index values for yoghurt are typically lower than those reported for milk (Table 1), which may be due to a number of reasons. For stirred yoghurt, it has been shown that not only the whey proteins, but also the caseins are subject to rapid gastric emptying [67], presumably because they are not susceptible to coagulation in the stomach [100,101]. As a result, the caloric proportion of carbohydrate in the emptied digesta is likely further reduced. Furthermore, during yoghurt fermentation, part of the lactose is hydrolyzed into glucose and galactose and it is subsequently primarily the produced glucose that is further fermented into lactic acid, whereas the galactose remains largely unmetabolized [102]. Hence, the glycemic potential of the carbohydrate fraction in yoghurt is lower than in milk due to the higher galactose:glucose ratio in the carbohydrate fraction in yoghurt. A further aspect that may affect the glycemic response to yoghurt consumption is the fact that lactate was recently shown to reduce the rate of gastric emptying in humans [103]. This may also contribute to glycemic control after yoghurt consumption. Overall, it is clear from the above that dairy products, such as milk and yoghurt, enable the delivery of carbohydrate, in the form of lactose, with accurate control over blood glucose levels though a multi-facetted control mechanism of gastric emptying and lactose digestion, supplemented by the low GI of one of the monosaccharides, i.e., galactose. However, some of these unique features are not present in other food matrices, as discussed in Section 5 for plant-based drinks, which are often positioned as dairy alternatives.

## 5. Glycemic Responses to Plant-Based Drinks Positioned as Dairy Alternatives

In recent years, there has been a growing interest worldwide in plant-based drinks positioned as milk alternatives for various reasons [7,104,105,106,107]. These plant-based drinks are water extracts of plants (legumes, oilseeds, cereals or pseudocereals) [108,109] positioned to be alternatives to milk and, therefore, typically developed with the aim of being quite similar to bovine milk in terms of appearance, texture, taste and shelf life, then to be used for similar applications [108,110]. Globally, soy drink is the most common plant-based milk alternative [7,108]. In addition to soy, other plant ingredients, such as oats, almonds, coconuts, rice and quinoa, can be used to produce plant-based drinks [7,108,109,111]. Moreover, plant-based drinks are usually fortified with one or more nutrients to make them nutritionally similar to bovine milk [107,108,112]. In addition to being cheaper, dairy products have a higher content of protein and essential amino acids, energy, fat, potassium, phosphorus and zinc than dairy alternative drinks [8,107,108,113]. Most of these plant-based drinks are characterized by a lower bioavailability of minerals and vitamins than milk, mainly due to the presence of antinutrient compounds, but also due to the fact that they are not fully dissolved in the water leaving sedimentation in the bottom, so if not shaken before use, then very little of the vitamins and minerals is ingested [8,107,108,111,112].

As for the total carbohydrate content, the average in bovine semi-skimmed milk is about 4.7 g lactose/100 g, while, for plant-based drinks, it is about 1.5 (coconut drink), 2.1 (soy drink), 2.7 (almond drink), 4.4 (oat drink) and 5.7 (rice drink) g of free sugar/100 g [113]. In the plant-based drinks, most sugars are classified as free sugars, which are described by the WHO [114] as all sugars (all monosaccharides and disaccharides) that have been added to beverages or food products by the manufacturer, cook or consumer, and those naturally occurring in fruit juices, syrups and honey and fruit juice concentrates, often associated with higher body weight and also with dental caries [114]. Lactose in milk is naturally present and, thus, not classified as a free sugar [107,113]. The main carbohydrates present in plant-based drinks positioned as milk substitutes are glucose, sucrose, maltose and fructose [7,8]. These sugars (except fructose) are known to have high-glycemic-index values compared to those of lactose [30,115].

Table 2 shows the nutritional composition and glycemic properties of different plant-based milk substitutes from in vivo and in vitro studies. For instance, a study by Vogelsang-O’Dwyer et al. [116] determined, through an in vitro method, the glycemic index of blue and white lupine milk alternatives, formulated to contain similar levels of protein and fat as low-fat bovine milk. The glycemic index of lupin drinks was compared with that of bovine milk (low fat) and soy drink, bought at a local supermarket (used as reference products). Bovine milk showed the lowest glycemic index (GI = 45), followed by the blue lupine (GI = 51) and white lupine (GI = 54) drinks. Instead, the soy drink showed the highest estimated glycemic index (GI = 58). The higher estimated glycemic index of the soy drink may be due to the presence of starch, which leads to the release of glucose during digestion [116]. It should be noted, however, that effects are calculated mainly based on carbohydrate composition and gross composition and do not include consideration of specific food matrix effects moderating rates of digestion and gastric emptying, which are highly relevant, as discussed earlier. Furthermore, Vogelsang-O’Dwyer et al. [116] calculated the glycemic load and found a higher glycemic load in bovine milk than soy drink and lupine drinks, attributed to the higher amount of carbohydrates.

Jeske et al. [7] estimated the in vitro glycemic index of seventeen various commercial plant-based drinks made from several cereals, nuts and legumes. The results showed that bovine milk had the lowest glycemic index (GI = 47) followed by calcium-enriched organic soy drink (GI = 48). The rest of the plant-based milk alternatives had a glycemic index between 49 and 64, with the exception of rice drinks and coconut drink, which showed very high estimated GI values, even up to 100. The high estimated glycemic index in these plant-based drinks may be due to the added sugars or sweeteners [7].

Table 2 shows that the rice drinks with high glycemic index also have a high GL value (18.33 and 16.85), due the high amount of carbohydrates, whereas other plant-based drinks showed low to moderate GL values. Anafy et al. [88] analyzed the glycemic indices of the bovine milk protein formula, a lactose-free formula and a soy-protein-based formula in twenty healthy adult volunteers (between 25 and 40 years old) and no significant differences in the glycemic index among the three formulas were found, although postprandial glucose peak was significantly higher after consumption of the soy-protein-based formula.

Pineli et al. [117] analyzed the glycemic index of a quinoa drink developed with high amounts of protein (by adapting the same process used for rice drink extraction) and compared it with those of bovine milk and a commercial rice drink. The results showed that the quinoa drink had a lower glycemic index than the rice drink (52 vs. 79) [117]. The higher amount of protein in quinoa milk may have contributed, in part, to its lower glycemic index, which delays digestion and gastric emptying [118]. Furthermore, the difference found in glycemic indices between quinoa and rice drinks may also be due to the starch granules, which vary between different food sources, or to the presence of 20-hydroxyecdysone (the most common phytoecdysteroid found in quinoa seeds), known to lower blood glucose levels and increase insulin sensitivity [117,119,120]. However, bovine milk still had the lowest glycemic index, followed by quinoa drink and rice drink (39, 52 and 79, respectively). Atkinson et al. [40] reported the average glycemic index of 62 common foods (obtained from multiple studies of various laboratories), including dairy alternatives, such as soy drink and rice drink. The authors observed a low GI for the soy drink (GI = 34), similar to that of bovine milk (GI = 39 for full-fat milk and GI = 37 for skim milk) and a high GI for the rice drink (GI = 86). Both soy drink and bovine milk have been shown to reduce the glycemic response of the meal through different mechanisms [121]. Sun et al. [121] reported that the consumption of bovine milk is associated with higher blood levels of branched-chain amino acids and GLP-1, probably responsible for the reduction in the glycemic response by delaying gastric emptying, while consumption of soy drink has instead been correlated with higher concentrations of non-essential amino acids (alanine, arginine and glycine) and GIP, probably involved in hyperinsulinemia.

Overall, from the studies discussed in this section, it is clear that in terms of glycemic responses, plant-based drinks positioned as milk alternatives may behave very differently than milk and milk products. Carbohydrate type and content will have a large effect and, while solely from a GI perspective, fructose may be preferable, the intense sweetness of fructose will limit application. Furthermore, while much is known about glycemic responses to dairy products and factors controlling it, including the role of milk components in gastric emptying, such knowledge is currently lacking for plant-based drinks positioned as dairy alternatives. With type 2 diabetes among the major diet-induced non-communicable diseases worldwide, such insights appear crucial to prevent inadequate messaging to consumers. From these insights, as well as other points highlighted in previous studies [8,107,113], a call for nutritional standards for plant-based drinks positioned as milk alternatives, as published by Drewnowski et al. [6], appears warranted. However, as outlined in the Introduction, such standards may require the inclusion of qualitative aspects for not only protein but also carbohydrate, to avoid risks of a diet-related increased disease burden.

## 6. Conclusions and Future Perspectives

While milk has a long history of safe consumption and use within the human diet, plant-based drinks, which are positioned as milk alternatives, have been a much more recent introduction into many diets. As outlined in this paper, glycemic responses following consumption of these products vary widely and can also vary substantially from milk. Such differences can be attributed to compositional differences, but also to specific matrix effects for the milk matrix, which will add adequate control of postprandial blood glucose levels. For plant-based drinks, such effects have received only limited attention to date but should be considered to avoid excessive glycemic responses following consumption.

## Figures and Tables

**Table 1:** Nutritional composition and glycemic properties of different dairy products.

Foods	Experimental Portion § (g or mL)	GI	II	GL (Per Serving)	Standard Serving Size * (g)	Carbohydrate (g/100 g)	Available Carbohydrate (g)	Sugar	Protein (g)	Fat (g)	Subjects (n)	Subjects Type	References
Milk, full fat		39											
Milk, skim	DNS	37	DNS	DNS	DNS	DNS	DNS	DNS	DNS	DNS	DNS	DNS	[40]
Ice cream		51											
Yoghurt, fruit		41											
Milk, skimmed, pasteurised, British	500 g	48		6	250	5.0	12.5 (g per serving)				10		
Milk, semi-skimmed, pasteurised, British	500 g	25		3	250	5.0	12.5 (g per serving)				8		
Milk, semi-skimmed, pasteurised, organic	500 g	34	DNS	4	250	5.0	12.5 (g per serving)	DNS	DNS	DNS	9	Healthy subjects	[85]
Milk, standardised homogenised, pasteurised, British	531.9 g	46		5	250	4.7	11.8 (g per serving)				10		
Milk, whole, pasteurised, fresh, organic	520.8 g	34		4	250	4.8	12.0 (g per serving)				9		
Yoghurt, low fat, natural	431 g	35		4	200	5.8	11.6 (g per serving)				9		
Whole milk	DNS	41	148	DNS	DNS	DNS	25	DNS	DNS	DNS	9	Healthy subjects	[86]
Skimmed milk		37	140				25				8		
Regular milk		30	90				25		17.3	15.3	10		
Fermented milk (Filmjölk)	DNS	15	98	DNS	DNS	DNS	25	DNS	19.2	17.4	10	Healthy subjects	[87]
Fermented milk (Ropy milk)		15	97				25		19.3	17.0	10		
Lactose		68	50				24		DNS	DNS	10		
Ice cream		36					50				5		
Milk (skim)		32					50				6		
Milk (whole)	DNS	34	DNS	DNS	DNS	DNS	50	DNS	DNS	DNS	6	Healthy subjects	[30]
Yoghurt		36					50				5		
Milk, full-cream condensed, sweetened	90.2 g	61	DNS	DNS	DNS	DNS	55.4 (g/100g)	DNS	DNS	DNS	11	Healthy subjects	[38]
Bovine milk protein- based formula (whey 60%, casein 40%)	200 mL	21.5	DNS	DNS	DNS	Lactose (100%)	50 (g per experimental portion)	DNS	9 (g per experimental portion)	DNS	20	Healthy subjects	[88]
Lactose-free formula (whey 60%, casein 40%)	200 mL	21.5				Glucose syrup solids (100%)	50 (g per experimental portion)		9 (g per experimental portion)		20		

* Standard serving size is the amount of food generally served, not the amount used for GI testing but for GL calculation; §Experimental portion is the amount of food used for GI testing; DNS, data not supplied; GI, glycemic index; II, insulinemic index; GL, glycemic load.

**Table 2:** Nutritional composition and glycemic properties of different plant-based milk substitutes.

Foods	Experimental Portion § (mL)	GI	II	GL (Per Portion)	Portion Size * (g or mL)	Carbohydrate (g)	Available carbohydrate (g)	Sugar (g/100 g)	Protein (g/100 g)	Fat (g/100g)	Subjects (n)	Subjects Type	References
Bovine milk, full fat		39											
Bovine milk, skim	DNS	37	DNS	DNS	DNS	DNS	DNS	DNS	DNS	DNS	DNS	DNS	[40]
Soy milk		34											
Rice milk		86											
Bovine milk	DNS	39				4.80	DNS	5.05	3.15	3.25	DNS		
Quinoa milk	312.5	52	DNS	DNS	DNS	14.7	50 (g per experimental portion)	9.7	1.7	0.2	12	Healthy subjects	[117]
Rice milk	DNS	79				12.7	DNS	7.3	0.30	1.11	DNS		
Bovine milk protein-based formula (whey 60%, casein 40%)	200	21.5				Lactose (100%)	50 (g per experimental portion)		9 g (g per experimental portion)		20		
Soy protein-based formula (soy 100%)	200	29.1	DNS	DNS	DNS	Glucose syrup solids (100%)	50 (g per experimental portion)	DNS	9 g (g per experimental portion)	DNS	20	Healthy subjects	[88]
Lactose-free formula (whey 60%, casein 40%)	200	21.5				Glucose syrup solids (100%)	50 (g per experimental portion)		9 g (g per experimental portion)		20		
Bovine milk		44.66		5.08	250 mL		0.5	Lactose (4.55 g/100 mL)	3.83%	1.28%			
Blue lupin milk alternative		51.15		2.98	250 mL		0.5	Sucrose (2.33 g/100 mL)	3.54%	1.43%			
White lupin milk alternative	DNS	53.71	DNS	3.06	250 mL	DNS	0.5	Sucrose (2.28 g/100 mL)	3.44%	1.37%	In vitro	In vitro	[116]
Soy milk alternative		57.73		3.76	250 mL		0.5	Sucrose (2.43 g/100 mL) and raffinose/stachyose (0.189 g/100 mL)	3.20%	1.69%			
Almond MLK		58.68		0.94	250 g		0.5	0.58	2.11	4.40			
Almond original		49.10		4.60	250 g		0.5	3.69	0.41	1.18			
Organic almond drink		64.21		0.37	250 g		0.5	0.16	0.95	3.69			
Carob almond MLK		54.33		6.32	250 g		0.5	4.58	2.4	3.35			
Organic cashew drink		52.82		4.76	250 g		0.5	2.87	0.87	2.50			
Coconut original		96.82		4.81	250 g		0.5	1.86	0.08	0.84			
Hazelnut original	DNS	55.76	DNS	4.37	250 g	DNS	0.5	3.09	0.36	1.56	In vitro	In vitro	[7]
Hemp milk unsweetened		59.94		0.21	250 g		0.5	0.09	0.08	2.44			
Organic macadamia drink		49.47		3.71	250 g		0.5	2.79	0.29	2.62			
Organic oat drink		59.61		7.98	250 g		0.5	3.35	0.70	0.38			
Quinoa drink		53.28		4.51	250 g		0.5	3.2	0.22	2.32			
Organic rice drink natural		97.74		18.33	250 g		0.5	7.02	0.32	0.85			
Organic brown rice drink		99.96		16.85	250 g		0.5	5.58	0.07	0.95			
Organic soya drink, calcium		47.53		3.01	250 g		0.5	2.43	2.72	2.11			
Plain UHT organic soya drink		54.02		1.24	250 g		0.5	0.88	3.70	2.04			
Soya organic, wholebean		49.49		0.57	250 g		0.5	0.36	3.16	1.77			
Soya original		61.50		4.87	250 g		0.5	3.09	2.61	1.48			
Fresh milk, pasteurised & homogenised		46.93		4.03	250 g		0.5	3.38	3.70	3.28			

* Portion size is the amount of food generally served, not the amount used for GI testing but for GL calculation; §Experimental portion is the amount of food used for GI testing; DNS, data not supplied; GI, glycemic index; II, insulinemic index; GL, glycemic load.

## Data Availability

No new data were created or analyzed in this study. Data sharing is not applicable to this article.
